# Attachable Hydrogel Containing Indocyanine Green for Selective Photothermal Therapy against Melanoma

**DOI:** 10.3390/biom10081124

**Published:** 2020-07-29

**Authors:** Juyoung Hwang, Jun-O Jin

**Affiliations:** 1 Shanghai Public Health Clinical Center, Shanghai Medical College, Fudan University, Shanghai 201508, China; jyhwang5@yu.ac.kr; 2 Department of Medical Biotechnology, Yeungnam University, Gyeongsan 38541, Korea; 3 Research Institute of Cell Culture, Yeungnam University, Gyeongsan 38541, Korea

**Keywords:** hydrogel, melanoma, photothermal therapy, indocyanine green (ICG), near-infrared (NIR) laser

## Abstract

Melanoma is the most lethal form of skin cancer because it spreads easily to other tissues, thereby decreasing the efficiency of its treatment via chemo-, radio-, and surgical therapies. We suggest the application of an attachable hydrogel for the treatment of melanoma whereby the size and amount of incorporated indocyanine green (ICG) for photothermal therapy (PTT) can be controlled. An attachable hydrogel (poly(acrylamide-co-diallyldimethylammonium chloride); PAD) that incorporates ICG as a near-infrared (NIR) absorber was fabricated using a biocompatible polymer. The temperature of PAD-ICG increases under 808 nm laser irradiation. The hydrogel protects the ICG against decomposition; consequently, PAD-ICG can be reused for PTT. The attachment of PAD-ICG to an area with melanoma in mice, with irradiation using a NIR laser, successfully eliminated melanoma. Thus, the data suggest that PAD-ICG is a smart material that could be used for selective target therapy against melanoma in humans.

## 1. Introduction

Melanoma is the deadliest form of skin cancer, as it can spread easily on skin or metastasize from skin to other tissues, such as the mouth, intestines, and eyes [1,2]. Melanoma remains an important threat to health, with metastasis often resulting in death [3]. Melanoma is typically treated by surgical removal during the early stages, but the excision of skin by surgery is difficult for large lesions, such as on the face, toes, and fingers [4]. Other approaches to treat melanoma include chemotherapy and radiotherapy, which can cause pain and harm to the body. A major limitation of chemotherapy and radiotherapy is their unpleasant side effects, and an inability to specifically target the melanoma cells [5,6]. Moreover, melanoma is the most investigated cancer, with a defined specific antigen for therapeutic vaccines. However, it is difficult for immune cells on infiltrate skin tissue due to a lack of blood supply in comparison with other peripheral tissues [7]. Therefore, advanced and alternative therapeutic treatments are required for the removal of melanoma. 

Photothermal therapy (PTT) has received extensive attention as an alternative therapeutic measure against cancer. Near-infrared (NIR) light is converted into heat-generated thermal energy through an NIR absorber. The specific delivery of an NIR absorber to the tumor cells is one strategy for photothermal therapy against cancer [8]. Because the NIR irradiation of absorbance-delivered cancer cells containing delivered absorbers elicits cell death by apoptosis and necrosis, it can reduce undesirable side-effects such as damage to healthy tissues [9]. Indocyanine green (ICG) is a water-soluble anionic tricarbocyanine dye with NIR absorption properties when exposed to 808 nm laser irradiation [10,11]. It has been used to enhance the local photothermal effect as an NIR absorber [12,13]. It has been approved as an NIR clinical imaging agent by the Food and Drug Administration (FDA) in the USA and the European Medicine Agency (EMA) for human use [14]. Although ICG is a suitable candidate for a photothermal therapeutic agent, it has several drawbacks, such as a fast degradation, low solubility, and poor stability in the body [9]. Various nanocarriers have been introduced to encapsulate ICG and overcome these limitations.

For anti-cancer therapy, numerous types of polymeric hydrogels have been developed [15,16,17,18]. There are several advantages to using a hydrogel as a carrier for the delivery of anti-cancer drugs and hydrophobic PTT agents, such as a good biocompatibility, high stability, and controlled drug release [19,20,21]. Most trials on hydrogels for the treatment of cancer have investigated the injection of hydrogels into mice [22,23,24]. A series of injectable and cross-linked hydrogels using biocompatible and carbohydrate-based polymers such as quaternized chitosan, hyaluronic acid, fucoidan, and poly-ethylene glycol have been developed as nanocarriers for the delivery of therapeutic agents [25,26,27,28,29,30,31,32]. However, hydrogels remain in the injection site for a certain period of time, which may promote inflammation and other side effects [33,34].

Because melanoma is a skin cancer and rapidly spreads into the surrounding skin, we aimed to determine whether melanoma can be treated using attachable hydrogels along with PTT. The hydrogels were synthesized using poly (acrylamide-*co*-diallyldimethylammonium chloride) (PAD) through the free radical polymerization of acrylamide (AM), *N*,*N*’-methylenebisacrylamide (BisAA), and diallyldimethylammonium chloride (DADMAC) in the presence of a cross-linking agent. The PAD hydrogels incorporated with ICG (PAD-ICG), which can be attached to the skin and induce PTT against melanoma under NIR laser irradiation.

## 2. Materials and Methods

### 2.1. Reagents and Materials

*N*,*N*,*N*’,*N*’-tetramethylethylenediamine (TEMED, 99.0%), *N*,*N*’-methylenebisacrylamide (BisAA, 99.5%), and ammonium persulfate (APS), IR-806, silicon 2,3-naphthalocyanine dichloride (SND), 5,10,15,20-tetraphenyl-21H, 23H-porphine (TPP) were acquired from Sigma Aldrich (Saint-Louis, MI, USA). The reagent DADMAC (65.0 *wt*.% in water) and ICG were obtained from Tokyo Chemical Industry (TCI, Tokyo, Japan). An acrylamide/Bis solution (19:1, 30 *wt*.% in water) was purchased from Bio-Rad (Hercules, CA, USA). All the materials were used without further purification, and all the solutions were diluted in deionized water.

### 2.2. Preparation of Poly(Acrylamide-co-Diallyldimethylammonium Chloride) Hydrogels

PAD, PAD-0.2ICG, and PAD-0.5ICG hydrogels, which contained 0, 0.2, and 0.5 mg/mL of ICG, respectively, were prepared by radical polymerization. The PAD hydrogels were produced by the following methods described in the published reports with some modifications [35]. The compositions of the PAD formulations are shown in Table 1. First, 1.5 mL of 30% AM, 0.75 mL of 60 *w/w*% DADMAC, 0.75 mL of 1.4 *w/w*% BisAA, 0.038 mL of 10 *w/v*% APS, 0.038 mL of TEMED, and 0.7 mL of deionized water (DW) were added under gentle stirring until the individual components were no longer visible. The solution was introduced into a circular mold, and the mixture was then placed in a thermostatic oven at 30 °C for 20 min to achieve a polymerization reaction to form the hydrogel. This was followed by cutting the gel into the same sized pieces, and then staining it with a standard dye solution (ICG, TPP, IR806 and SND) with stirring at 60 rpm at 4 °C overnight. Finally, the hydrogels were transferred into DW for 24 h to remove any soluble impurities.

### 2.3. Determination of Indocyanine Green Oncentration in Poly (Acrylamide-co-Diallyldimethylammonium Chloride) Hydrogels

The loaded concentration of ICG in the PAD hydrogels was determined by using the remaining ICG standard solution after the ICG staining. The concentration of ICG unloaded into each PAD hydrogel was measured using a UV–vis spectrophotometer (Cary 100 Bio, Varian Inc., Palo Alto, CA, USA). The loading efficiency of ICG in the PAD hydrogels was determined to be 99.24% for 0.2 mg/mL of ICG, 99.23% for 0.5 mg/mL.

### 2.4. Characterization

Fourier transform spectroscopy (FTIR; Spectrum 100, Perkin Elmer, Waltham, MA, USA) was used to examine the IR spectrum of PAD. Scanning electron microscopy (SEM) images were taken using an S-4800 scanning electron microscope (HITACHI, Ibaraki, Japan). A fiber-coupled continuous-wave diode laser (808 nm, 10 W) was purchased from Changchun New Industries Optoelectronic Technology Co., Ltd. (Changchun, China). Thermographic images and changes in temperature were measured using a FLIR ONE (FLIR Systems, Wilsonville, OR, USA).

### 2.5. Apoptosis Assay

Cells were stained with annexin V-fluorescein isothiocyanate (FITC) and 4′,6-diamidino-2-phenylindole (DAPI) (Sigma-Aldrich, St. Louis, MI, USA) in 100 μL of binding buffer (BioLegnd, San Diego, CA, USA) for 15 min at room temperature (RT). The cells were analyzed by flow cytometry using a NovoCyte flow cytometer (ACEA Biosciences Inc., San Diego, CA, USA) after 400 μL of binding buffer was added without washing. For active caspase 3 staining, the cells were fixed and permeabilized with Cytofix/Cytoperm buffer (eBioscience, San Diego, CA, USA) and subsequently incubated with anti- active caspase 3 Abs in Perm/Wash buffer (BioLegnd) for 30 min. Staining of the isotype control immunoglobulin (Ig) G was performed in all the experiments. The active caspase 3 expression levels were analyzed by NovoCyte flow cytometer (ACEA Biosciences Inc.). 

### 2.6. Near-Infrared Photothermal Heating

A fiber-coupled continuous-wave diode laser (808 nm, 10 W) was purchased from Changchun New Industries Optoelectronics Technology Co., Ltd. Thermographic images and changes in temperature were captured using a FLIR ONE (FLIR Systems).

### 2.7. Mice

C57BL/6 mice (6–8 weeks old) were obtained from Korea Orient Bio Co. Inc. (Seongnam, Korea). The mice were kept under pathogen-free conditions at the Laboratory Animals Center of Yeungnam University. All experiments were conducted after considering the basic ethical principle of animal experiments and the 3R principles (replacement, reduction and refinement). In addition, the experiment was conducted in compliance with the animal protection law, the law on experimental animals, and the Institutional Animal Care and Use Committee regulations of Yeungnam University. The committee on the Ethics of Animal Experiments of Yeungnam University Laboratory Animals Center approved the protocol (Mouse Protocol Number, 2019-040). In accordance with the humanitarian end point criteria, for ethical reasons, we tried to minimize the pain or stress of the animals by euthanizing them with CO_2_ gas.

The murine melanoma cell line B16 (from American Type Culture Collection, Manassas, VA, USA) and human melanoma cell line A375P and A375SM (both from Korean Cell Line Bank, Seoul, Korea) were cultured in DMEM (Sigma Aldrich) and supplemented with 10% FBS, 2 mM glutamine, 1 M *N*-(2-hydroxyethyl)piperazine-*N*′-(2-ethanesulfonic acid), 100 μg/mL of streptomycin, 100 U/mL of penicillin, and 2 mM 2-mercaptoethanol. The cell line was cultured at 37 °C in a humidified atmosphere consisting of air enriched with 5% CO_2_.

### 2.8. Melanoma Photothermal Therapy

Once the tumor size reached approximately 5.0 mm in the longest dimension on day 5, the mice were randomly placed into three groups based on the treatment strategies used: no PAD, PAD, and PAD-0.2ICG with laser irradiation. After each of the hydrogels were attached to the mice, the tumors were irradiated using an 808 nm NIR laser with a power density of 1 W/cm^2^ for 5 min. The temperature was captured by an infrared camera (FLIR Systems).

### 2.9. Hematoxylin and Eosin Staining

Lung, liver and colon samples were fixed in 4% paraformaldehyde, embedded in paraffin, and sectioned to 5 μm thickness. Sections were then stained with hematoxylin and eosin (H&E), and examined for tissue damage.

### 2.10. Melanoma Photothermal Therapy

Data are expressed as the mean ± standard error of the mean. The *p*-value was analyzed using SigmaPlot software from Systat Software Inc. (San Jose, CA, USA), and values of <0.05 were considered to have statistical significance.

## 3. Results

### 3.1. Preparation and Characterization of Poly (Acrylamide-co-Diallyldimethylammonium Chloride) Hydrogels 

**Figure 1:**
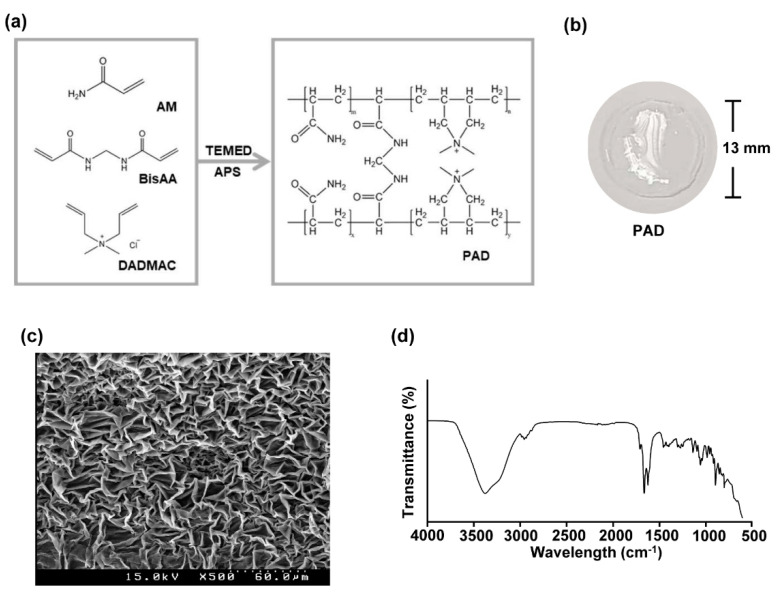
Structure and characterization of the PAD hydrogel: (**a**) synthetic scheme of the PAD hydrogel, (**b**) photograph of the PAD hydrogel, and (**c**) the SEM image of the lyophilized PAD hydrogel, and (**d**) the FTIR spectrum of the PAD hydrogel.

The free radical polymerization of AM and DADMAC monomers was used for preparation of PAD hydrogels, which were cross-linked with BisAA along with APS/TEMED to catalyze the polymerization. During polymerization at 30 °C, the initiator APS was dissociated in sulfated anion radicals in the presence of TEMED. After a radical initiator was formed, the copolymerization of the AM and DADMAC occurred, along with a reaction with BisAA as the cross-linker (Figure 1a). The compositions of the pre-gel solution are shown in Table 1. A photograph of a PAD hydrogel with a diameter of approximately 13 mm is shown in Figure 1b. As shown in Figure 1c, the PAD hydrogels were lyophilized for the observation of the inner structure using SEM. The SEM images show a cross-section of a PAD hydrogel with a typical microporous structure. As shown in Figure 1d, the chemical structure of PAD-ICG was analyzed by FT-IR spectroscopy. The amide bond in the AM moiety presents at 1620 cm^−1^ and the peak of 1451 cm^−1^ was attributed to the quaternary amine belonging to DADMAC. In addition, the position of the sharp band at 1134 cm^−1^ evidenced the presence of the secondary amine from the cross-linker (BisAA) portion. Therefore, it was concluded that a PAD hydrogel can form an interconnected pore scaffold, revealing an excellent reaction with the reactants.

### 3.2. Preparation and Characterization of Poly (Acrylamide-co-Diallyldimethylammonium Chloride) Hydrogels

**Figure 2:**
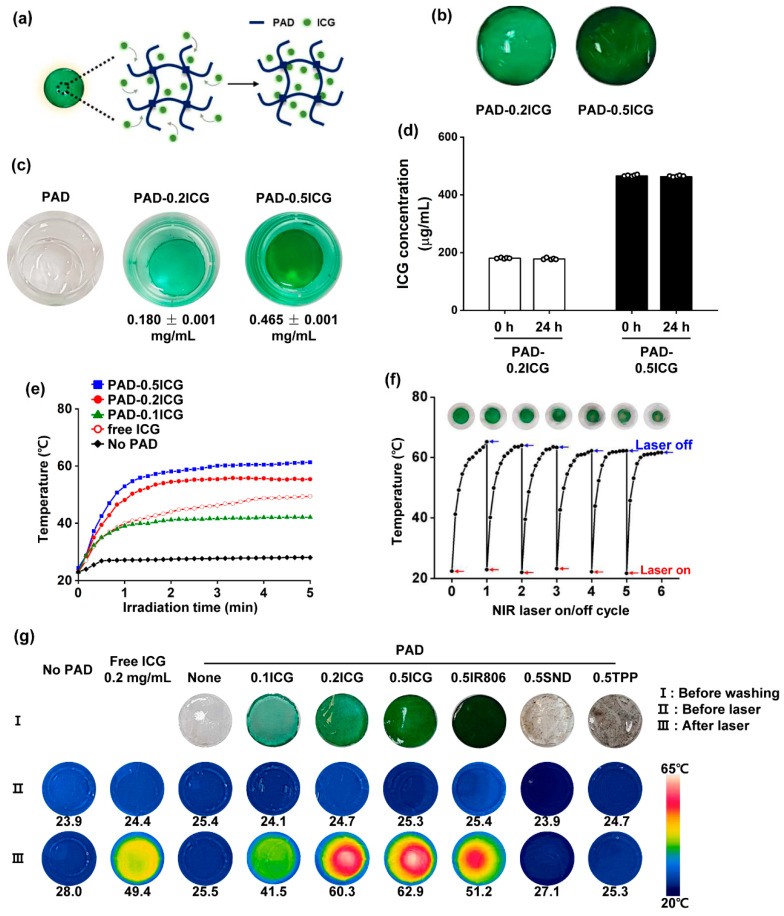
Characterization of the PAD-indocyanine green (ICG) hydrogel: (**a**) the illustration of the PAD-ICG structure; (**b**) the photograph of the PAD hydrogel loaded with 0.2 mg/mL ICG (PAD-0.2ICG) and 0.5 mg/mL ICG (PAD-0.5ICG); (**c**) the photograph of PAD after ICG staining overnight; (**d**) the ICG concentration in the PAD hydrogel after ICG staining; (**e**) the temperature variation curve of 0.2 mg/mL ICG and the different concentrations of PAD-ICG under 808 nm laser irradiation for 5 min with a laser power density of 1 W/cm^2^; (**f**) the temperature variation curve of the PAD-0.2ICG hydrogel for six cycles of consecutive laser irradiation of 5 min with a power density of 1 W/cm^2^; and (**g**) the temperature and camera imaging of the indicated dye-incorporated PAD hydrogel. The indicated numbers are the temperature of the PAD hydrogels after irradiation at 1 W/cm^2^ for 5 min.

Based on the free radical polymerization of soluble AM and a DADMAC polymer, 0.2 and 0.5 mg/mL of ICG were incorporated into the PAD hydrogels, as indicated in the methods section (Figure 2a). As shown in Figure 2b, the PAD-0.2ICG and PAD-0.5ICG hydrogels were successfully fabricated and incorporated into ICG. PAD-0.2ICG and PAD-0.5ICG hydrogels were successfully fabricated and incorporated into ICG. The concentration of ICG in PAD-ICG was determined by the difference between the total quantity and the outer solution concentration using UV–vis absorbance after staining. The loading efficiency of PAD-0.2ICG and PAD-0.5ICG were 90.46% and 93.13%, which corresponded to 0.180 ± 0.001 mg/mL and 0.465 ± 0.001 mg/mL, respectively (Figure 2c). To determine the stability of the PAD-ICG in aqueous solutions, the PAD hydrogels were placed in a phosphate buffer saline solution (PBS) for 24 h. PAD-0.2ICG and PAD-0.5ICG released 0.76% and 0.77% of the ICG, respectively (Figure 2d).

To evaluate the photothermal efficiency, we measured the temperature change under laser irradiation (1 W/cm^2^) at 808 nm for 5 min, and found notable increases in the temperature for PAD containing ICG compared to pure PAD. The PAD-0.2ICG rapidly reached maximum temperatures within the first 1.5 min. In addition, the increase of the temperature of the PAD-0.2ICG with laser irradiation was higher than that of 0.2 mg/mL of free equivalent ICG with laser irradiation. (see Figure 2e and Table 2). To evaluate the photothermal stability of PAD-ICG, the temperature change of PAD-0.2ICG under irradiation with a 1 W/cm^2^ laser at 808 nm for 5 min (with the laser on, as indicated by the red arrows) was monitored. It was then cooled to RT for six repeated cycles (with the laser off, as indicated by the blue arrows). Although there were slight decreases in the temperature following repeat NIR irradiation, the PAD maintained the ICG’s color and gel formation after the three counts of NIR irradiation. (Figure 2f). We also examined whether the PAD hydrogel can be incorporated with other dyes, and loaded 0.5 mg/mL of IR-806, SND, and TPP on the PAD hydrogel (PAD-0.5IR806, PAD-0.5SND, and PAD-0.5TPP, respectively). The maximum temperature of PAD-0.5IR806 increased by 51.2 °C (Δ 25.8 °C) under NIR laser irradiation of 1 W/cm^2^ for 5 min. However, the temperatures of the PAD hydrogels incorporated with SND and TPP, which feature a low solubility of the aqueous solution, did not increase with NIR irradiation (Figure 2g). Thus, these results indicate that PAD-0.2ICG and PAD-0.5ICG can be considered promising photothermal materials.

### 3.3. Poly (Acrylamide-co-Diallyldimethylammonium Chloride) and Laser Irradiation Induced Cell Death of Melanoma Cell Lines

**Figure 3:**
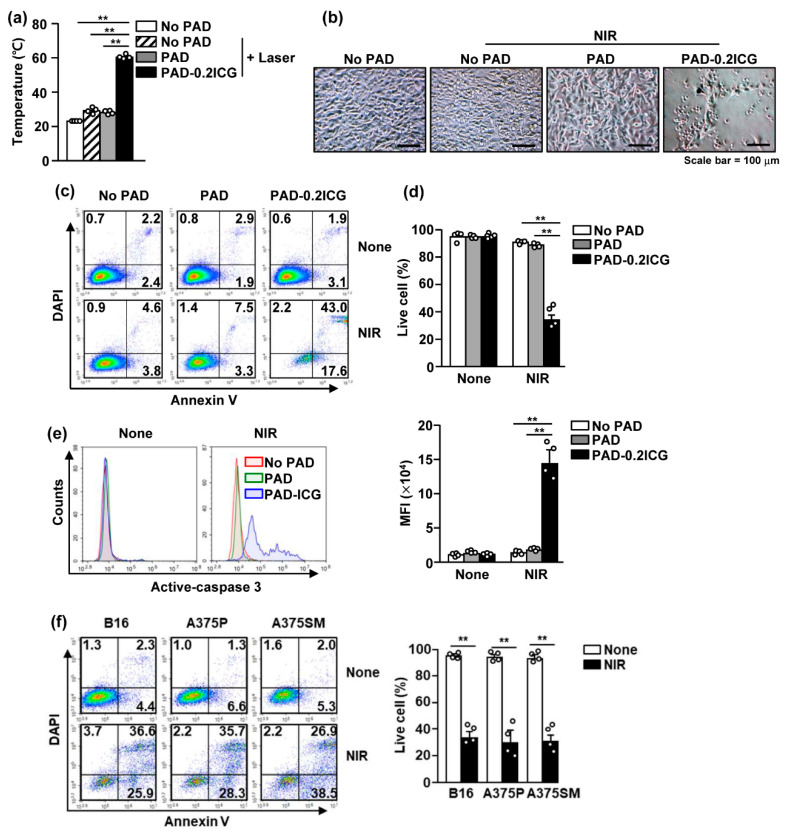
In vitro photothermal therapy (PTT) by PAD-ICG hydrogel against melanoma cells. B16 cells were treated with PAD-0.2ICG and irradiated with near-infrared (NIR) laser (1 W/cm^2^ for 5 min): (**a**) the average temperature of pure PAD and PAD-0.2ICG after 808 nm laser irradiation at 1 W/cm^2^ for 5 min; (**b**) the cell morphology was shown 24 h after the laser irradiation; (**c**) the cell apoptosis and necrosis were analyzed by annexin-V and 4′,6-diamidino-2-phenylindole (DAPI) staining; (**d**) the mean percentage of annexin-V^-^ and DAPI^-^ live cells was shown; (**e**) the intracellular active-caspase 3 levels measured by flow cytometry (left panel) and the mean fluorescence intensity (MFI) of active-caspase 3 are shown; and (**f**) B16, A375P and A375SM were treated with PAD-ICG and irradiated with NIR at 1 W/cm^2^ for 5 min. The apoptotic and necrotic cells were analyzed by annexin-V and DAPI staining (left panel). Mean live cells were shown (right panel). Data are representative or show the average of four independent samples (two independent experiments performed with *n* = 2/group, two-way ANOVA, mean ± SEM), ** *p* < 0.01.

Because NIR irradiation in PAD-ICG effectively increased the temperature, we then examined whether PAD-ICG and laser irradiation promotes the apoptosis and necrosis of melanoma cells. NIR irradiation in PAD-0.2ICG-treated wells dramatically increased the temperature, while the temperature was not considerably changed in other controls with NIR laser irradiation (Figure 3a). PAD-0.2ICG with laser irradiation on B16 cells promoted substantial decreases in the attachment of B16 cells compared to the other controls (Figure 3b). The number of live B16 cells decreased remarkably when using PAD-0.2ICG with NIR irradiation compared to other controls (Figure 3b–d). In addition, active caspase 3, a marker of apoptotic cells, was significantly increased when using PAD-0.2ICG and laser irradiation (Figure 3e). We also examined whether PAD and NIR irradiation can induce the cell death of human melanoma cells. The results indicated that the apoptotic and necrotic cell populations of A375P (a malignant human melanoma cell line) and A375SM (a highly metastatic human melanoma cell line) cells increased substantially (Figure 3f). Thus, these data indicate that PAD-ICG and laser irradiation can induce the apoptosis and necrosis of mouse and human melanoma cells.

### 3.4. Poly (Acrylamide-co-Diallyldimethylammonium Chloride) Hydrogels and Laser Irradiation Eliminated Melanoma by Photothermal Therapy

**Figure 4:**
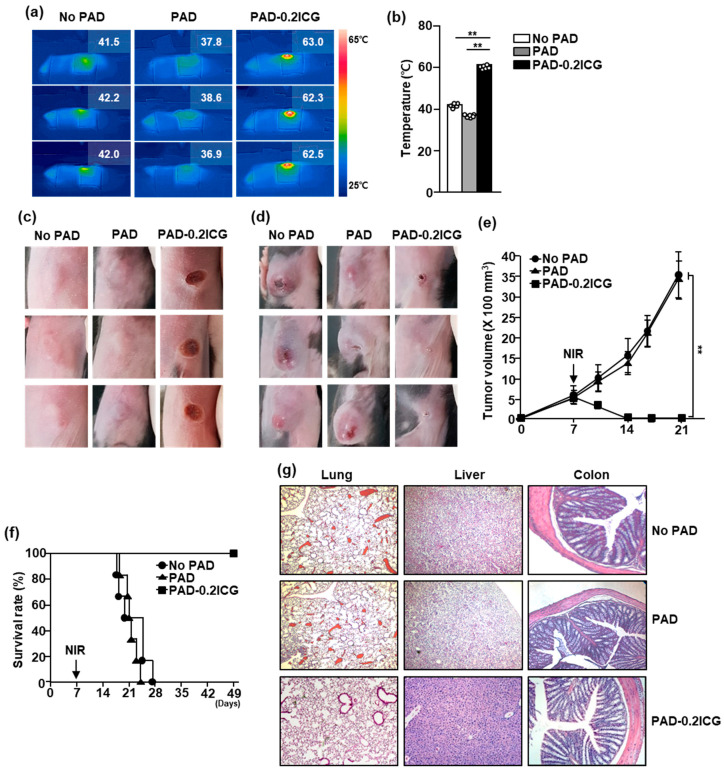
In vivo anti-melanoma therapeutic effect of PAD-ICG hydrogel using photothermal therapy. C57BL/6 mice were subcutaneously (*s.c.*) inoculated with 1 × 10^6^ B16 cells: (**a**) NIR temperature camera imaging corresponding to the temperature distribution of PAD hydrogel loaded with 0.2 mg/mL of ICG. The temperature changes of pure PAD and PAD-ICG after 808 nm laser irradiation with 1 W/cm^2^ for 5 min are shown; (**b**) the average temperature of pure PAD and PAD-ICG after irradiation at 1 W/cm^2^ for 5 min. B16 tumor masses on day 10 (**c**) and on day 17 (**d**) after B16 tumor inoculation are shown; (**e**) the B16 tumor growth curves are shown; (**f**) on day 21 after tumor injection, the mice were sacrificed and the lung, liver and colon were harvested and the tissues stained with hematoxylin and eosin (H&E); and (**g**) the survival rates of mice were shown. Data are representative or show the average of six independent samples (two independent experiments performed with *n* = 3/group). Significance was determined by log-rank test, ** *p* < 0.01 (*n* = 6).

Because laser irradiation increased the temperature of PAD-ICG, we then examined whether it could be used in the treatment of B16 melanoma in mice. Once the B16 tumor mass was established on day 7, PAD or PAD-0.2ICG was attached on the top of the tumor mass and irradiated with an 808 nm NIR laser at 1 W/cm^2^ for 5 min (Figure 4a). The NIR laser irradiation induced the marked increases in temperature of up to 61.4 ± 2.42 °C in the PAD-0.2ICG attached on the tumor mass, whereas the pure PAD reached 38.3 ± 1.31 °C with laser irradiation (Figure 4b). Three days after laser irradiation was applied, the color of the tumor mass changed to black, indicating that burning had occurred (Figure 4c). The melanoma tumor in the mice had nearly disappeared on day 10 after PTT, owing to the attachment of PAD-0.2ICG and NIR laser irradiation (Figure 4d). As shown in Figure 4e, the size of the B16 tumor decreased rapidly when using PAD-0.2ICG and NIR laser irradiation. Moreover, no PAD and PAD with laser-irradiated mice died within 28 days after tumor injection, while the PAD-0.2ICG with laser irradiation protected the mice till 50 days after tumor challenge (Figure 4f), and tumor recurrence was not confirmed within 50 days after tumor injection. In addition, the attachment of PAD and PAD with laser irradiation in the skin did not promote any inflammation or wound in the skin (Figure S1). Moreover, the tissue inflammation or complications in the peripheral tissues were not observed in the mice treated with PAD-ICG and laser irradiation (Figure 4g). Therefore, these results suggest that the laser irradiation of attachable PAD-0.2ICG can be used to treat melanoma.

## 4. Discussion

Melanoma is a type of skin cancer that is difficult to treat through surgery. It readily spreads to the adjacent skin, and therefore, it is difficult to perform targeted therapy for this cancer. In this study, we fabricated PAD hydrogels incorporated with ICG. The temperature of the PAD hydrogels could be efficiently increased with irradiation by an 808 nm laser, and they were used as a treatment for melanoma in mice. The hydrogels were fabricated using water-soluble polymers, including AM, BisAA, and DADMAC, in the presence of a cross-linking agent, due to which the PAD hydrogels were mechanically stronger than the injectable hydrogels. Moreover, the PAD hydrogels were attachable to skin owing to their insoluble and elastic properties. The hydrogels could also be cut into various sizes, and this property is extremely effective for the treatment of widespread melanoma in the skin. Thus, PAD hydrogels will become a therapeutic candidate for the treatment of melanoma. 

Because PAD-ICG have a micro-sized porous structure, ICG can be incorporated into these hydrogels, allowing a response to irradiation from an NIR laser. Due to the concentration of the ICG staining solution, the osmotic pressure differs between the interior PAD and the exterior ICG solution, following the ICG’s absorption into the PAD. Additionally, anionic ICG penetrates the polymeric network of PAD while interacting with its quaternary ammonium groups. Therefore, 0.2 and 0.5 mg/mL of ICG were incorporated into PAD, although the thermal responses of PAD-0.2ICG and PAD-0.5ICG did not differ substantially. The reason for the low differential in the responses of PAD-0.2ICG and PAD-0.5ICG against an NIR laser may be due to the absorptivity of ICG. Numerous studies have shown that ICG is unable to promote dose-dependent increases in temperature in response to an NIR laser. This may be due to a saturation of the absorptivity of ICG with regard to a NIR laser [36]. 

Although ICG is a wellknown NIR absorber, it degrades easily after NIR irradiation and cannot be protected against photobleaching under NIR irradiation. We also found that PAD-ICG responds to NIR lasers for at least three repeated cycles. PAD-ICG’s reusability may be due to ICG’s protective properties against photobleaching, as shown by a lack of change in the PAD’s color. Moreover, PAD-ICG responds more powerfully against NIR laser irradiation compared to free ICG. In the next study, we will investigate how PAD protects against decomposition.

Treatment with polymer-based injectable hydrogels are a very interesting method under development in cancer therapy. The injectable hydrogels demonstrated the effective delivery of anti-cancer drugs to a tumor mass and promoted the therapeutic effect against cancer in mice. In addition, ICG-incorporated injectable hydrogels have also shown the promising effect of PTT against tumors [37,38]. Despite their biodegradability, however, inflammation may develop at the injected site because degradation takes several days [39]. Moreover, spreading skin melanoma cannot be targeted using injectable hydrogels. To overcome these undesirable aspects, we suggest using an attachable version. Because PAD is not injected in vivo, it does not promote inflammation in the tissue. Moreover, a flexible, size-controllable PAD that is appropriate for targeting the spread of melanoma in skin should be developed.

## 5. Conclusions

Melanoma is the deadliest skin cancer, as it can easily spread to the adjacent skin and other tissues. In this study, we synthesized PAD-ICG for selective attachment to melanoma and to enable the irradiation of tumors using a NIR laser for PTT. The PAD-ICG only targeted attached tissues and successfully eliminated melanoma in the skin. Thus, PAD-ICG is applicable for the treatment of melanoma in humans.

## Figures and Tables

**Table 1:** Composition ratio of the PAD (poly (acrylamide-*co*-diallyldimethylammonium chloride) hydrogel.

Compositions	Volume (mL)
Deionized water (DW)	0.700
Acrylamide (AM, 30%)	1.500
Diallyldimethylammonium chloride (DADMAC, 60 *w/w*%)	0.750
*N*,*N*’-methylenebisacrylamide (BisAA, 1.4 *w/v*%)	0.750
Ammonium persulfate (APS, 10 *w/v*%)	0.038
*N*,*N*,*N*’,*N*’-tetramethylethylenediamine (TEMED)	0.038

**Table 2:** Changes in the temperature during 808 nm laser irradiation with a power density of 1 W/cm^2^ in PAD-ICG.

ICG conc. in PAD (mg/mL)	Temperature (°C)						
	0 m	1 m	2 m	3 m	4 m	5 m	ΔT
**0**	23.0	27.2	27.5	27.8	28	28.1	5.1
**0.1**	23.0	39.1	41.2	41.6	42	42.1	19.1
**0.2**	23.1	48.4	54.5	55.4	55.7	55.4	32.3
**0.5**	24.4	52.9	58.1	60.1	60.5	61.3	36.9

## Supplementary Materials

The following are available online at https://www.mdpi.com/2218-273X/10/8/1124/s1, Figure S1: Cytotoxicity to skin cells via No PAD and PAD hydrogel attachment with or without NIR laser irradiation.
