# Supplementary material for: Attachable Hydrogel Containing Indocyanine Green for Selective Photothermal Therapy against Melanoma
Source: Biomolecules. 2020 Jul 29;10(8):1124. doi: 10.3390/biom10081124 (PMC7465476; doi:10.3390/biom10081124)
Supplement: Supplementary file 1 [file biomolecules-10-01124-s001.pdf]

Supporting Information

Attachable hydrogel containing indocyanine green for selective photothermal therapy against melanoma

*Juyoung Hwang, and Jun-O Jin**

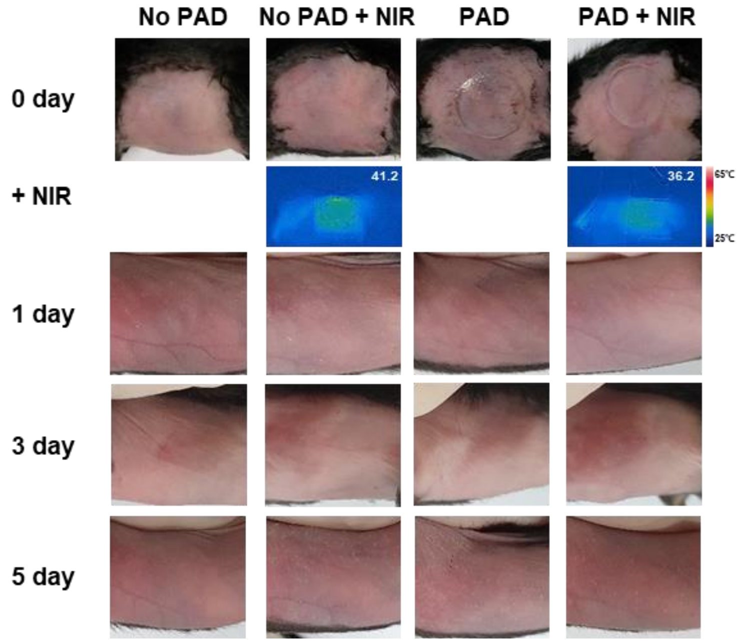

Figure S1. Cytotoxicity to skin cells via No PAD and PAD hydrogel attachment with or without NIR laser irradiation.
